# Maternal Viral Infection and Risk of Fetal Congenital Heart Diseases: A Meta‐Analysis of Observational Studies

**DOI:** 10.1161/JAHA.118.011264

**Published:** 2019-04-18

**Authors:** Ziwei Ye, Lesan Wang, Tubao Yang, Lizhang Chen, Tingting Wang, Letao Chen, Lijuan Zhao, Senmao Zhang, Zan Zheng, Liu Luo, Jiabi Qin

**Affiliations:** ^1^ Department of Epidemiology and Health Statistics Xiangya School of Public Health Central South University Hunan China

**Keywords:** case‐control study, congenital heart disease, infection, meta‐analysis, virus, Congenital Heart Disease, Meta Analysis, Risk Factors, Women

## Abstract

**Background:**

At present, the association between maternal viral infection and risk of congenital heart diseases (CHD) in offspring is uncertain; additionally, a complete overview is missing. A meta‐analysis of observational studies was performed to address the question of whether women who had a history of viral infection in early pregnancy were at an increased risk of CHD in offspring, compared with mothers without viral infection.

**Methods and Results:**

Unrestricted searches were conducted, with an end date parameter of July 15, 2018, of PubMed, Embase, Google Scholar, Cochrane Libraries, and Chinese databases, to identify studies that met prestated inclusion criteria. Seventeen case‐control studies involving 67 233 women were included for analysis. Both fixed‐effects models (odds ratio [OR], 1.83; 95% CI, 1.58–2.12; *P*<0.0001) and random‐effects models (OR, 2.28; 95% CI, 1.54–3.36; *P*<0.0001) suggested that mothers who had a history of viral infection in early pregnancy experienced a significantly increased risk of developing CHD in offspring. For specific viral infections, the risk of developing CHD in offspring was significantly increased among mothers with rubella virus (OR, 3.49, 95% CI, 2.39–5.11 in fixed‐effects models; and OR, 3.54; 95% CI, 1.75–7.15 in random‐effects models) and cytomegalovirus (OR, 3.95; 95% CI, 1.87–8.36 in fixed‐effects models) in early pregnancy; however, other maternal viral infections in early pregnancy were not significantly associated with risk of CHD in offspring. Sensitivity analysis yielded consistent results. No evidence of publication bias was observed.

**Conclusions:**

Although the role of potential bias and evidence of heterogeneity should be carefully evaluated, the present study suggests that maternal viral infection is significantly associated with risk of CHD in offspring.

**Clinical Perspective**

**What Is New?**

- Today, the association between maternal viral infection and risk of congenital heart disease in offspring is still uncertain; a complete review of the evidence to date has been lacking.
- Our study represents the first meta‐analysis of risk of congenital heart disease associated with maternal viral infections.

**What Are the Clinical Implications?**

- Our study shows that maternal viral infection, in particular infection with rubella or cytomegalovirus, in early pregnancy is significantly associated with risk of congenital heart disease in offspring, which indicates that early detection and intervention for viral infection may help to reduce the incidence of congenital heart disease.

Congenital heart disease (CHD) is the most common congenital birth defect. CHD accounted for nearly one third of all birth defects, and the prevalence rate reached to 8 to 12 per 1000 live births worldwide.^1^, ^2^ During neonatal period, the reported incidence of CHD was: Asia and Oceania, 10%; North America, 9%; Europe, 7%; South America, 6%; and Africa, 2%.^2^, ^3^ Additionally, the incidence of CHD in China was about 8.94%,^3^ ranking first in the number of birth defects detected in hospitals for many years.

Despite recent advances in medicine and surgery, CHD continues to cause much perinatal morbidity and mortality. It is reported that CHD caused ≈303 300 deaths in 2015, which was decreased from the 366 000 since 1990.^4^, ^5^ It has a great impact on the quality of life of children. In addition, the high cost of treatment puts heavy financial burden and mental pressure on families and society.

Several studies have demonstrated that a number of genetic and environmental factors have been associated with the development of CHD in the fetus. Furthermore, some risk factors have been identified, such as phenylketonuria, rubella, retinoic acid, and the use of certain specific drugs.^6^ Interestingly, there were some studies that indicated mothers infected with a virus during pregnancy may be at a higher risk of developing CHD in offspring.

However, available evidence to date has not found any consistent association between maternal viral infections and risk of CHD, and there are many conflicting results across previous studies. For example, some studies indicated that there is a weak or null association between maternal viral infections and CHD.^7^, ^8^, ^9^, ^10^, ^11^, ^12^, ^13^ In contrast, other studies found an increased risk of CHD among mothers infected with a virus during pregnancy.^14^, ^15^, ^16^, ^17^, ^18^, ^19^, ^20^, ^21^, ^22^, ^23^ Therefore, the relationship between maternal viral infections and risk of CHD remains controversial. Unfortunately, there still has not been a study published for systematic review and meta‐analysis on this topic. In the present study, we aimed to assess the relationship between maternal viral infections and risk of CHD in offspring through a meta‐analysis of cohort and case‐control studies.

## Materials and Methods

The authors declare that all supporting data are available within the article.

### Data Sources and Search Strategy

We attempted to report the present systematic review and meta‐analysis by following the recommendations of the Preferred Reporting Items for Systematic Reviews and Meta‐analyses statement.^24^ PubMed, Embase, Google Scholar, Cochrane Libraries, and Chinese databases (including China Biology Medicine disc, Chinese Scientific Journals Fulltext Database, China National Knowledge Infrastructure, and Wanfang Database) were searched through July 2018 with no restrictions, to identify studies that assessed the risk of CHD associated with maternal viral infection. The following search terms were used and combined: “(congenital heart disease OR congenital heart defect OR congenital heart malformation OR congenital heart anomalies OR congenital cardiac disease OR congenital cardiac defect OR congenital cardiac malformation OR congenital cardiac anomalies OR congenital cardiovascular disease OR cardiovascular malformation OR cardiovascular defect OR cardiovascular anomalies OR birth defect) AND (viral infection OR viral infection OR rubella virus OR cytomegalovirus OR hepatitis B virus OR coxsackie virus OR herpesvirus) AND (cohort studies OR prospective studies OR follow‐up studies OR retrospective studies OR case‐control studies).” We also performed a manual search on the reference lists of retrieved articles. Gray literature (generally refers to nonpublicly published literature) and conference abstracts were not searched. We did not contact authors of the primary studies for additional information.

### Inclusion Criteria

In this review, the exposures of interest were maternal viral infections. Mothers who had a history of viral infection, such as rubella virus, cytomegalovirus, hepatitis B virus, coxsackievirus, and herpesvirus, were defined as the exposed group, and those without viral infection as the unexposed group. The outcomes of interest were CHDs. We first performed an initial screening of titles or abstracts. A second screening was based on full‐text review. Studies were considered eligible if they (1) were published in Chinese or English; (2) had a cohort or case‐control design; (3) had use of maternal viral infection as the exposure of interest; (4) had use of CHDs as outcomes of interest; and (5) reported relative risks and odd ratios (ORs), with corresponding 95% CIs (or data to calculate them).

### Data Extraction

Data extraction was performed using a standardized data collection form. Any reported relative risks or ORs and their 95% CIs of CHD for mothers who had a history of viral infection compared with those without viral infection, as well as characteristics for each study, were extracted. Additionally, any reported relative risks or ORs and their 95% CIs describing the relationship between different types of viral infection and risk of CHD were also extracted. Corresponding information was recorded as follows: the first author's name, year of publication, geographic region, study design type, sample size, diagnostic methods of CHD, ascertainment of viral infection, viral infection time, reported types of viral infection, reported ORs and their 95% CIs, whether the confounding factors were adjusted when estimating the association between maternal viral infection and risk of CHD, and quality score.

### Quality Assessment

Two authors (Z.W.Y. and J.B.Q.) independently conducted the studies selection, data extraction, and quality assessment. Any disagreements were resolved through discussion among the authors until consensus was reached. We adopted the principles of the Newcastle–Ottawa Scale (available at http://www.ohri.ca/programs/clinical_epidemiology/oxford.asp) to assess the quality of included studies. In statistics, the scale is a tool used for assessing the quality of nonrandomized studies included in a meta‐analysis. Using the tool, each study is judged on 8 items, categorized into 3 groups: the selection of the study groups; the comparability of the groups; and the ascertainment of outcome of interest for cohort studies. Stars awarded for each quality item serve as a quick visual assessment. Stars are awarded such that the highest‐quality studies can be awarded as many as 9 stars. When the study gains ≥6 stars, it is considered of higher methodologic quality.

### Statistical Analysis

Statistical analysis was performed using Stata version 12.0 (StataCorp LP, College Station, TX) and Review Manager version 5.3 (The Nordic Cochrane Centre, The Cochrane Collaboration). Statistical tests were declared significant for a 2‐sided *P* value not exceeding 0.05, except where otherwise specified. Combined ORs and their corresponding 95% CIs were calculated by using fixed‐effect models and random‐effects models, to assess the association between maternal viral infections and risk of developing CHD in offspring. We focused not only on the relationship between maternal total viral infection and CHD in offspring but also on the relationship between specific viral infection and CHD in offspring. Homogeneity of effect size across studies was tested by using the Q statistics at the *P*<0.10 level of significance. The I^2^ statistic, which is a quantitative measure of inconsistency across studies, was also calculated (significance level at I^2^>50%).^25^, ^26^

Subgroup analyses were conducted according to study design type, ascertainment of viral infection, diagnostic method of CHD, whether confounding factors were controlled, and quality score. We further conducted a sensitivity analysis to examine the influence of various exclusion criteria on the overall risk estimate. We also investigated the influence of a single study on the overall risk estimate by omitting 1 study in each turn. Potential publication bias was assessed by using the Begg's funnel plots, the Begg's rank correlation test (significance level at *P*<0.10), and the Egger linear regression test (significance level at *P*<0.10). Subgroup analyses, sensitivity analyses, and publication bias assessment were performed only for the association between maternal total viral infection and risk of CHD in offspring because of rather small numbers of studies for specific viral infections.

## Results

### Literature Search

**Figure 1:**
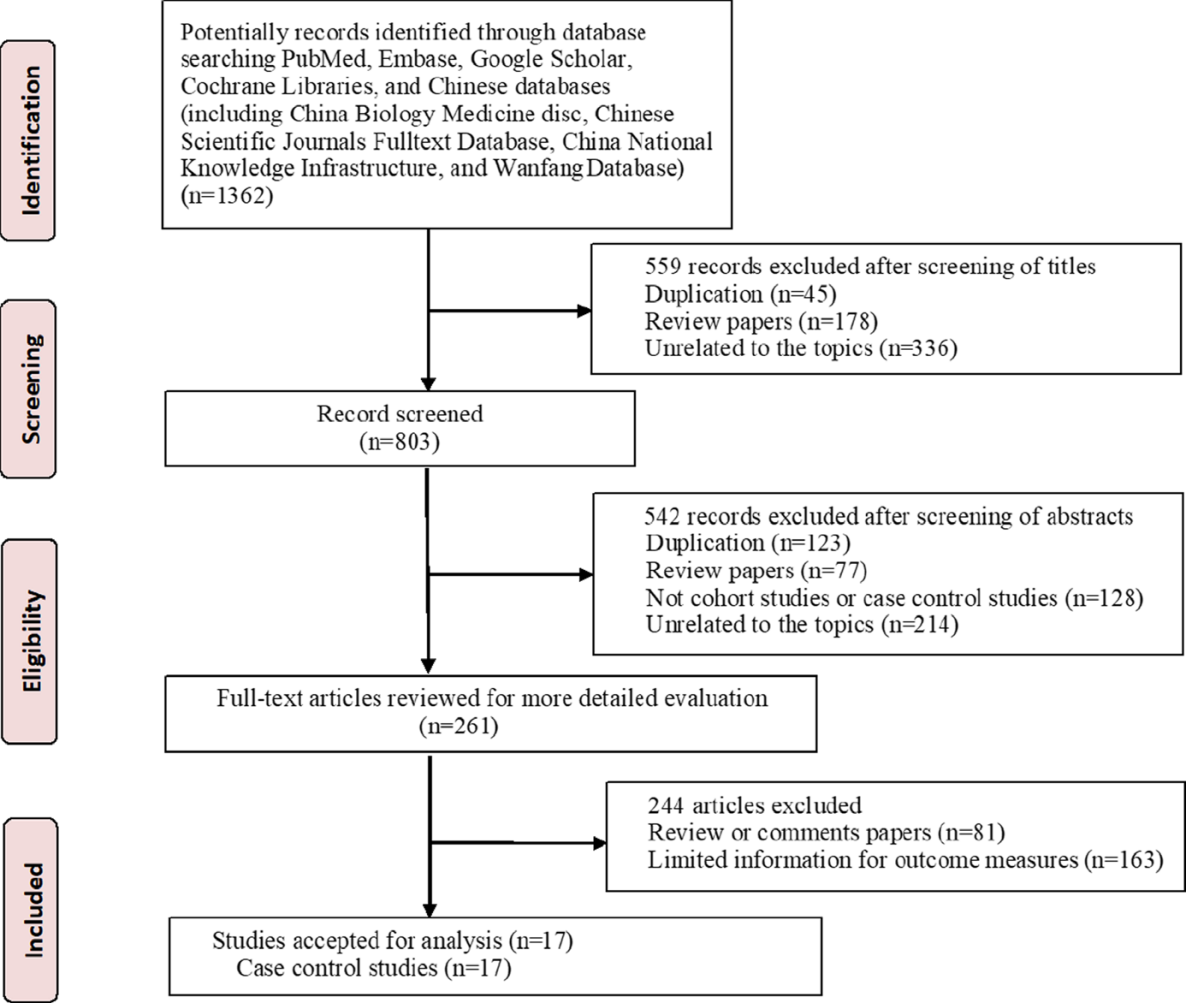
Flow chart showing the meta‐analysis studies selection.

We initially retrieved 1362 records from PubMed, Embase, Google Scholar, Cochrane Libraries, and Chinese databases. Of these, the majority were excluded after the first screening on the basis of abstracts or titles because of duplicates, reviews, missing original data, or unrelated to our analysis. After full‐text review of 261 studies, 244 studies that did not report the association between maternal viral infection and risk of CHD in offspring were further excluded. Finally, 17 case‐control studies^7^, ^8^, ^9^, ^10^, ^11^, ^12^, ^13^, ^14^, ^15^, ^16^, ^17^, ^18^, ^19^, ^20^, ^21^, ^22^, ^23^ were included for analysis (Figure 1).

### Characteristics of Included Studies

Characteristics of included studies, which involved 67 233 participants and were published between 1994 and 2018, are summarized in Table 1.^7^, ^8^, ^9^, ^10^, ^11^, ^12^, ^13^, ^14^, ^15^, ^16^, ^17^, ^18^, ^19^, ^20^, ^21^, ^22^, ^23^ Among the included studies, 16 studies (94.1%) were conducted in China and 1 (5.9%) in Canada. All studies were case‐control studies; 8 of the studies (47.1%) were 1:1 matched case‐control studies and 1 (5.9%) was a 1:2 matched case‐control study. Nine studies (52.9%) used laboratory testing to assess whether mothers were infected with a virus; while the remaining studies (47.1%) used questionnaires. In all studies included, the viral infection time was in the first trimester. Among the 17 studies included here, the number of studies reporting specific types of viral infection was as follows: 7 rubella virus; 6 herpesvirus; 4 cytomegalovirus; 4 hepatitis B virus; 3 coxsackievirus; and 3 other virus. Diagnosis of CHD was performed by ultrasound examination or surgery for all studies. All included studies were considered to be of higher methodologic quality, achieving a quality score of ≥6 of 9. However, only 6 studies (35.3%) controlled the confounding factors when assessing the association between maternal viral infection and risk of CHD in offspring.

**Table 1:** Characteristics of Included Studies

Author (Year of Publication)	Geographic Region	Study Design Type	Sample Size	Diagnosis Method of CHD	Ascertainment of Viral Infection	Viral Infection Time	Reported Types of Viral Infection	Reported ORs (95% CIs) of Specific Viral Infection	Reported ORs (95% CIs) of Total Viral Infection	Whether the Confounding Factors Were Adjusted (Yes/No)	Quality Score
Liu^7^ (1996)	China	1:1 case control	280	Ultrasound examination or surgery	Antibody testing	First trimester	RV HSV Coxsackie B3 Coxsackie B4	3.42 (1.62–5.24) 1.13 (0.37–1.89) 1.23 (0.79–1.92) 1.36 (0.87–2.14)	1.56 (0.21–1.19)	No	8
Lai^8^ (2001)	China	1:1 case control	1522	Not stated	Self‐report	First trimester	RV HSV CMV HBV	65.55 (0.0061–705 555) 65.46 (0.0008–5 675 814) 23.02 (0.0013–418 649) 1.1014 (0.4650–26 088)	7.33 (0.16–334.31)	No	7
Liu^9^ (2009)	China	1:2 case‐control	291	Ultrasound examination or surgery	Self‐report	First trimester	Not stated	Not applicable	0.27 (0.09–0.83)	No	7
Chen^10^ (2012)	China	Case‐control	630	Ultrasound examination	Self‐report	First trimester	Not stated	Not applicable	1.00 (0.06–16.11)	No	6
Li^11^ (2016)	China	1:1 case‐control	100	Ultrasound examination	Antibody testing	First trimester	HPV B19 RV HSV CMV HBV	4.50 (1.61–12.55) 5.41 (1.66–17.65) 0.77 (0.34–1.74) 1.74 (0.39–7.71) 2.04 (0.18–23.27)	2.03 (0.18–23.11)	No	8
Fung^12^ (2013)	Canada	Case‐control	1674	Ultrasound examination	Antibody testing	First trimester	RV Others virus	0.59 (0.07–4.96) 0.58 (0.28–1.21)	0.58 (0.29–1.16)	Yes	7
Liang^13^ (2017)	China	Case‐control	5381	Not stated	Self‐report	First trimester	Measles RV VZV HBV	4.03 (0.51–32.01) 1.71 (0.10–29.31) 1.09 (0.07–18.19) 1.38 (0.33–5.72)	1.78 (0.65–4.89)	No	7
Zou^14^ (2015)	China	1:1 case‐control	300	Ultrasound examination	Self‐report	First trimester	Not stated	Not applicable	5.08 (1.14–22.57)	Yes	8
Liu^15^ (1994)	China	1:1 case‐control	206	Ultrasound examination or surgery	Antibody testing	First trimester	CMV	4.42 (1.83–10.67)	4.42 (1.83–10.67)	No	8
Yang^16^ (1995)	China	1:1 case‐control	280	Ultrasound examination	Antibody testing	First trimester	RV HSV	3.42 (1.62–5.24) 0.90 (0.53–4.55)	1.49 (1.06–2.09)	No	8
Chen^17^ (2010)	China	1:1 case‐control	276	Ultrasound examination	Self‐report	First trimester	Not stated	Not applicable	3.00 (1.09–8.05)	No	7
Guo^18^ (2010)	China	Case‐control	367	Ultrasound examination	Antibody testing	First trimester	Coxsackievirus RV HSV CMV HBV	137.82 (1.04–18 317.76) 8594.28 (29.96–2 465 338.78) 2879.81 (8.22–1 009 431.21) 2963.97 (9.11–963 880.71) 1448.83 (6.37–329 730.35)	1434.84 (123.38–16 687.04)	Yes	6
Zhao^19^ (2011)	China	1:1 case‐control	836	Not stated	Self‐report	First trimester	Not stated	Not applicable	3.44 (1.53–7.72)	No	7
Yu^20^ (2012)	China	Case‐control	2049	Not stated	Antibody testing	First trimester	Not stated	Not applicable	4.30 (1.57–11.82)	No	7
Ou^21^ (2013)	China	Case‐control	5057	Ultrasound examination	Antibody testing	First trimester	Not stated	Not applicable	1.94 (1.37–2.75)	Yes	7
Chen^22^ (2016)	China	Case‐control	1009	Ultrasound examination	Self‐report	First trimester	Not stated	Not applicable	2.26 (1.18–4.34)	Yes	8
Zhang^23^ (2018)	China	Case‐control	46 975	Ultrasound examination	Antibody testing	First trimester	Not stated	Not applicable	3.82 (1.96–6.92)	Yes	8

CHD indicates congenital heart disease; CMV, cytomegalovirus; HBV, hepatitis B virus; HPV B19, human parvovirus B19; HSV, herpesvirus; OR, odds ratio; RV, rubella virus; VZV, varicella‐zoster virus.

### Maternal Total Viral Infection and Risk of CHD in Offspring

**Figure 2:**
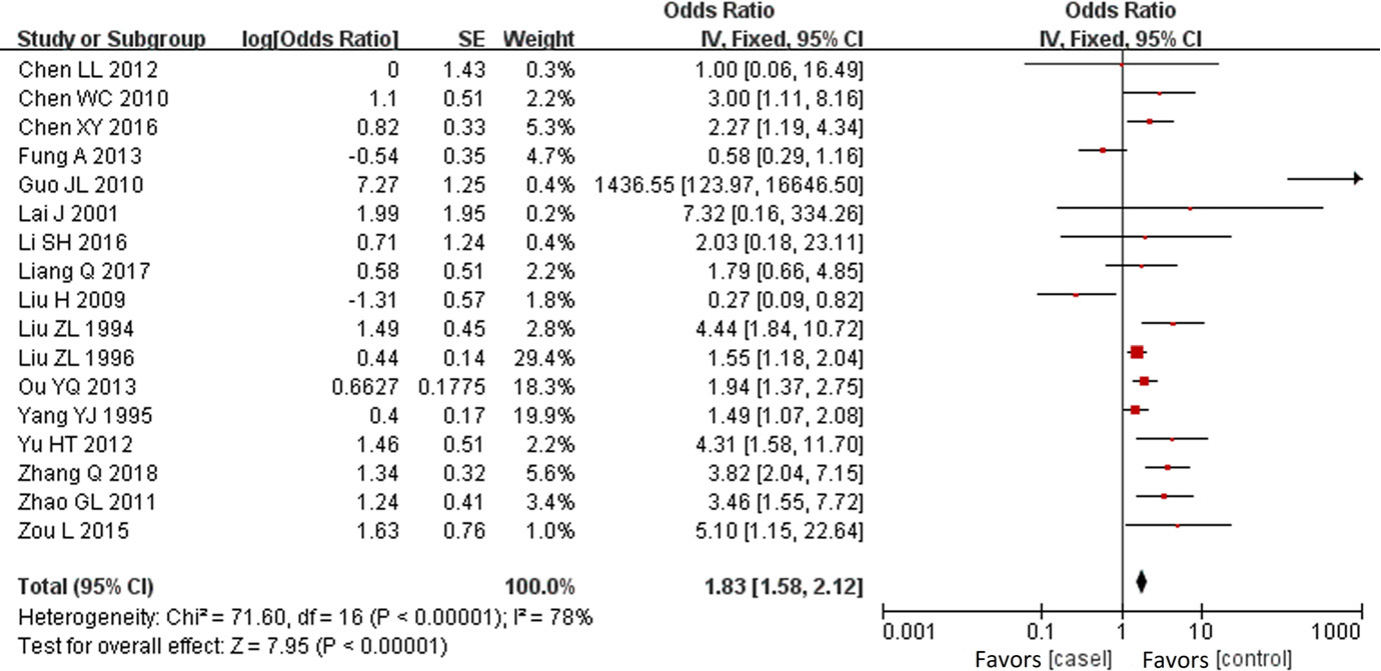
Forest plot of maternal viral infection and risk of CHD.^7^, ^8^, ^9^, ^10^, ^11^, ^12^, ^13^, ^14^, ^15^, ^16^, ^17^, ^18^, ^19^, ^20^, ^21^, ^22^, ^23^ The OR and horizontal lines represent the 95% CIs in fix‐effect model. CHD indicates congenital heart disease; OR, odds ratio.

**Figure 3:**
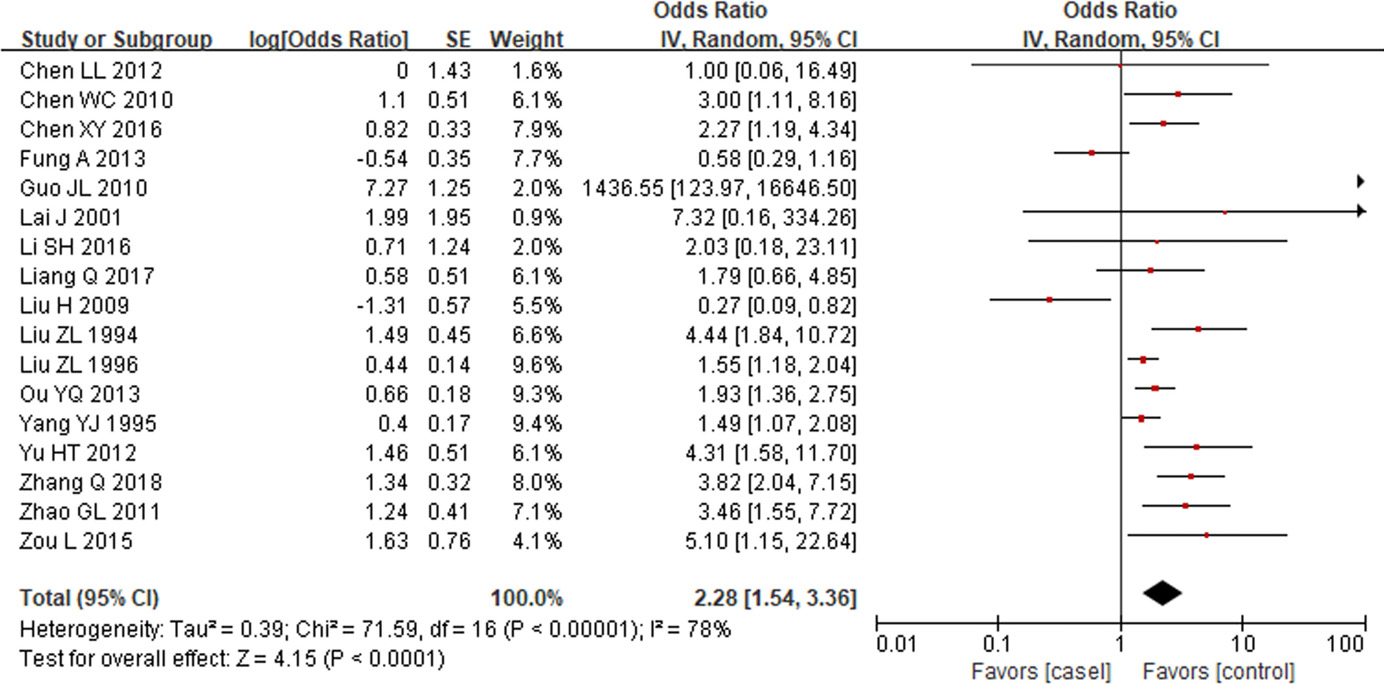
Forest plot of maternal viral infection and risk of CHD.^7^, ^8^, ^9^, ^10^, ^11^, ^12^, ^13^, ^14^, ^15^, ^16^, ^17^, ^18^, ^19^, ^20^, ^21^, ^22^, ^23^ The OR and horizontal lines represent the 95% CIs in random‐effect model. CHD indicates congenital heart disease; OR, odds ratio.

Both fixed‐effects models (OR, 1.83; 95% CI, 1.58–2.12; *P*<0.0001) and random‐effects models (OR, 2.28; 95% CI, 1.54–3.36; *P*<0.0001) suggested that mothers who had a history of viral infection in early pregnancy experienced a significantly increased risk of developing CHD in offspring when compared with those without viral infection (Figures 2 and 3).^7^, ^8^, ^9^, ^10^, ^11^, ^12^, ^13^, ^14^, ^15^, ^16^, ^17^, ^18^, ^19^, ^20^, ^21^, ^22^, ^23^ However, substantial heterogeneity (*P*<0.00001; I^2^=78%) was observed across studies.

### Maternal Specific Viral Infection and Risk of CHD in Offspring

**Figure 4:**
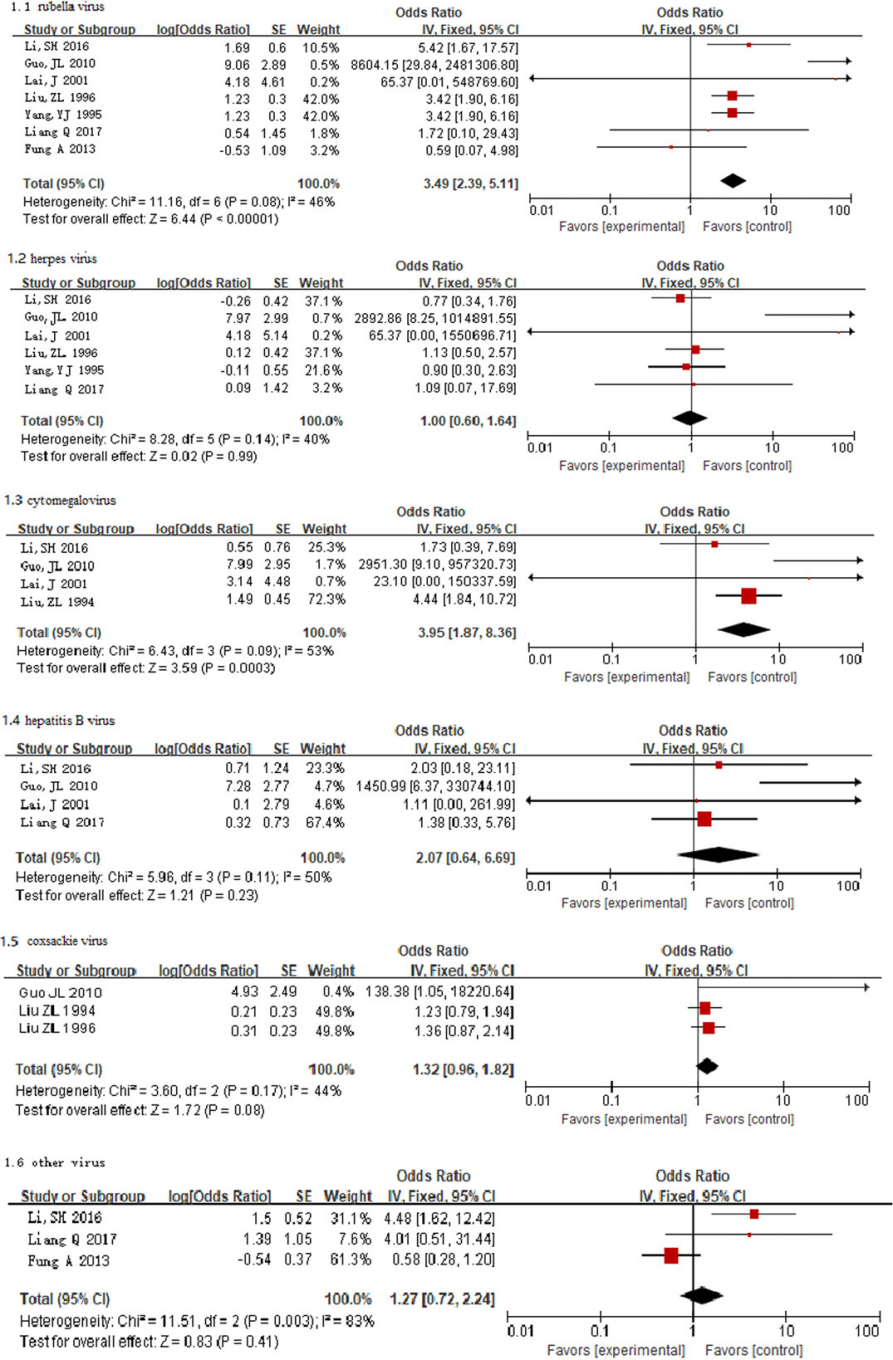
Forest plot of the specific viral infection and risk of CHD.^7^, ^8^, ^11^, ^12^, ^13^, ^15^, ^16^, ^18^ The OR and horizontal lines represent the 95% CIs in fixed‐effect model. CHD indicates congenital heart disease; OR, odds ratio.

Fixed‐effects models showed the risk of developing CHD in offspring was significantly increased among mothers with rubella virus (OR, 3.49; 95% CI, 2.39–5.11; *P*<0.00001) and cytomegalovirus (OR, 3.95; 95% CI, 1.87–8.36; *P*<0.001) in early pregnancy compared with the reference group. However, we did not find a significant association for other maternal viral infections (Figure 4).^7^, ^8^, ^11^, ^12^, ^13^, ^15^, ^16^, ^18^

**Figure 5:**
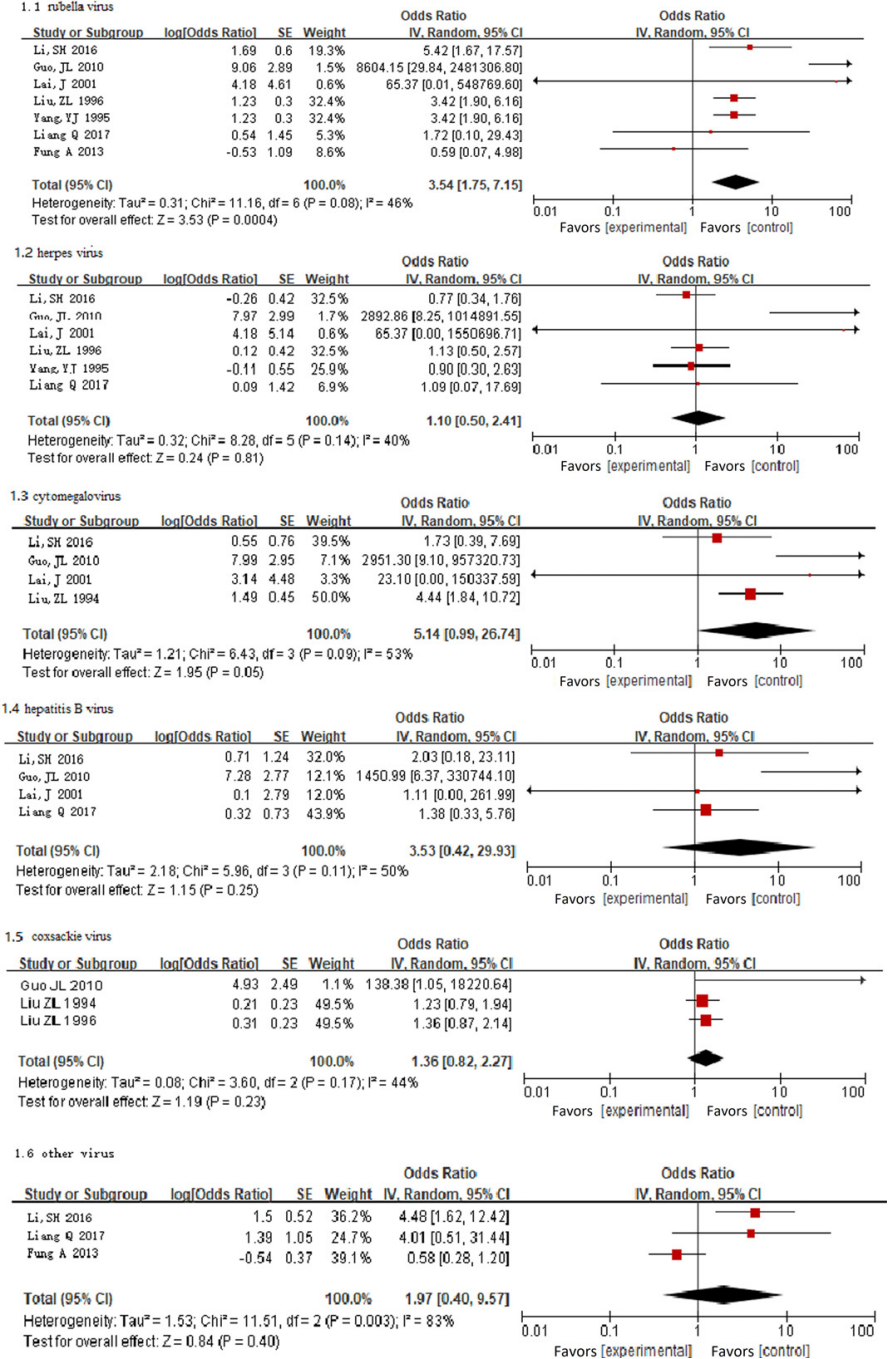
Forest plot of the specific viral infection and risk of CHD.^7^, ^8^, ^11^, ^12^, ^13^, ^15^, ^16^, ^18^ The OR and horizontal lines represent the 95% CIs in random‐effect model. CHD indicates congenital heart disease; OR, odds ratio.

Random‐effects models suggested that mothers infected with rubella virus in early pregnancy were at a significantly higher risk of developing CHD in offspring (OR, 3.54; 95% CI, 1.75–7.15; *P*=0.0004). However, other maternal viral infections in early pregnancy were not significantly associated with risk of CHD in offspring (Figure 5).^7^, ^8^, ^11^, ^12^, ^13^, ^15^, ^16^, ^18^ There was substantial heterogeneity for rubella virus (*P*=0.08; I^2^=46%), cytomegalovirus (*P*=0.09; I^2^=53%), and other virus (*P*=0.003; I^2^=83%).

### Subgroup Analyses

Subgroup analyses for the association between maternal total viral infection and risk of developing CHD in offspring were summarized in Table 2. After subgroup analysis, whether the confounding factors were adjusted (test for subgroup differences: I^2^=30.6%) was identified as the most relevant heterogeneity moderators. When data were restricted to adjusted studies (OR, 3.59; 95% CI, 1.45–8.90), the risk of developing CHDs increased further. However, there was no statistically significant difference for risk of CHD associated with viral infection between adjusted data and unadjusted data (χ^*2*^=1.44; *P*=0.23). Additionally, differences for risk of CHD associated with viral infection were not statistically significant between the remaining subgroups (all *P*≥0.38). Overall, mothers with viral infection were still at higher risk of CHD in offspring among most subgroup data except for lower‐quality studies.

**Table 2:** Subgroup Analysis for the Association Between Viral Infection and Risk of CHD

Subgroup Variables	No. of Studies	Pooled OR (95% CI)	Measure of Heterogeneity		
			χ^2^	*P* Value	I^2^
Study design type			0.77^a^	0.38^a^	0.0%^a^
Paired case‐control study	9	1.97 (1.26–3.07)	22.88	0.004	65.0%
Case‐control study	8	2.90 (1.38–6.09)	9.42	<0.001	85.0%
Ascertainment of viral infection			0.34^a^	0.56^a^	0.0%^a^
Self‐report	8	1.97 (1.03–3.77)	17.12	0.020	59.0%
Antibody testing	9	2.52 (1.51–4.21)	53.92	<0.001	85.0%
Diagnostic method of CHD			1.18^a^	0.28^a^	15.5%^a^
Ultrasound examination or surgery	13	2.12 (1.35–3.31)	65.49	<0.001	82.0%
Not stated	4	3.10 (1.83–5.25)	1.85	0.60	0.0%
Whether the confounding factors were adjusted			1.44^a^	0.23^a^	30.6%^a^
Adjusted	6	3.59 (1.45–8.90)	45.80	<0.001	89.0%
Unadjusted	11	1.08 (0.92–1.27)	24.27	0.007	59.0%
Quality score			1.35^a^	0.51^a^	0.0%^a^
=6	2	39.18 (0.03–48 647.21)	14.65	<0.001	93.0%
=7	8	1.66 (0.89–3.07)	28.43	<0.001	75.0%
=8	7	2.25 (1.57–3.22)	14.24	0.03	58.0%

CHD indicates congenital heart disease; OR, odds risk.

a Test for subgroup differences.

### Sensitivity Analyses

**Figure 6:**
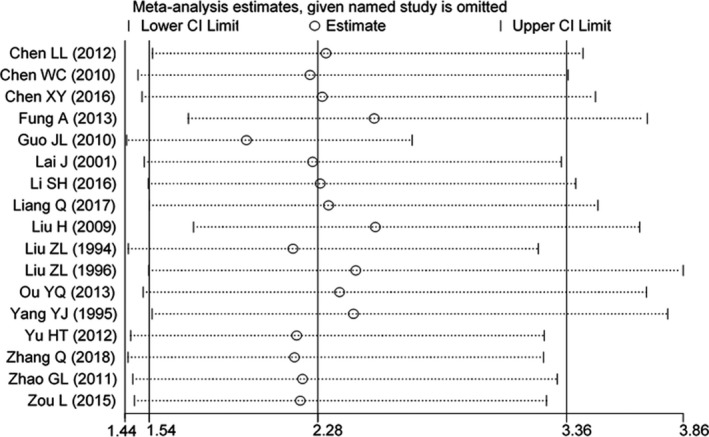
Sensitivity analyses.^7^, ^8^, ^9^, ^10^, ^11^, ^12^, ^13^, ^14^, ^15^, ^16^, ^17^, ^18^, ^19^, ^20^, ^21^, ^22^, ^23^

Sensitivity analyses were conducted to examine the influence of various exclusion criteria on the overall risk estimates. Exclusion of 11 studies that did not control any confounding factors when assessing the association between maternal viral infection and CHD showed a somewhat greater risk (OR, 3.59; 95% CI, 1.45–8.90), yet heterogeneity was still present (*P*<0.001; I^2^=89%). Exclusion of 13 studies in which the quality scores were <8 yielded similar results (OR, 2.25; 95% CI, 1.57–3.22), with substantial evidence of heterogeneity (*P*=0.03; I^2^=58%). Further exclusion of any single study did not materially alter the overall risk estimates (Figure 6).^7^, ^8^, ^9^, ^10^, ^11^, ^12^, ^13^, ^14^, ^15^, ^16^, ^17^, ^18^, ^19^, ^20^, ^21^, ^22^, ^23^

### Tests for Publication Bias

**Figure 7:**
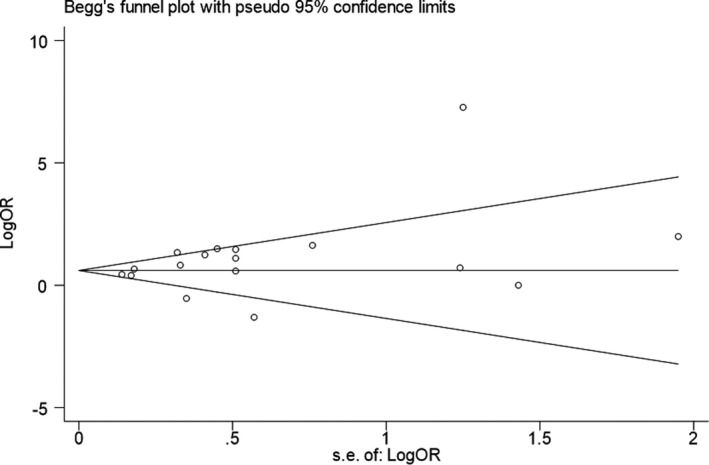
Funnel plot of maternal viral infection and risk of CHD. CHD indicates congenital heart disease.

Visual inspection of the Begg's funnel plot did not identify substantial asymmetry (Figure 7). Furthermore, the Begg's rank correlation test (Z=0.12; *P*=0.903) and the Egger linear regression test (t=−1.40; *P*=0.189) also indicated no evidence of publication bias among studies of maternal viral infection and CHD risk in offspring.

## Discussion

CHDs constitute the most common birth defects among newborns and have emerged as one of the most important causes of infant mortality. Additionally, CHDs have a significant impact on child and adult morbidity and disability.^27^, ^28^ Along with the increasing prevalence of CHD, an increasing number of researchers are interested in its etiology, but its etiology remains unclear. Our meta‐analysis of 17 case‐control studies involving 67 233 participants including 7720 mothers of CHD cases and 59 513 mothers of control infants, with sufficient statistical power, aimed at addressing the question of whether an increased risk of developing CHD exists in mothers who were infected with a virus in early pregnancy compared with those who were not infected with a virus. An improved understanding of this issue may have important public health and clinical implications, given the possibility that prevention and treatment of maternal viral infection might reduce the incidence of CHD in offspring.

Findings from the present meta‐analysis indicated that mothers infected with a virus in early pregnancy were at a significantly increased risk of developing CHD in offspring when compared with those without viral infection (OR, 1.83 for fixed‐effects models and 2.28 for random‐effects models). When data were restricted to adjusted studies (OR, 3.59), the risk of developing CHD increased further. For specific viral infections, our study showed that mothers with rubella virus and cytomegalovirus in early pregnancy had a higher risk of developing CHD in offspring compared with mothers without viral infections. However, our review did not indicate that other maternal viral infections, including herpes virus, hepatitis B virus, and coxsackievirus, significantly increased the risk CHD in offspring. Of note, these results must be viewed with caution because of some evidence of heterogeneity (I^2^ range, 40%–83%).

A series of case‐control studies have been performed to assess the association between maternal viral infections and risk of CHD in offspring, but the results were often inconsistent. For example, Chen et al,^17^ Chen et al,^22^ Liu et al,^7^, ^15^ Zhang et al,^23^ and Zhao et al^19^ reported that maternal viral infections significantly increased risk of CHD in offspring; but other studies did not find statistically significant differences between mothers with viral infection and those without viral infection for the risk of CHD, and 1 study^9^ even reported a significantly decreased risk of CHD. For rubella virus, most studies^7^, ^11^, ^16^, ^18^ showed a significantly positive association, and several studies^8^, ^12^, ^13^ reported a weak or null association. For herpesvirus, cytomegalovirus, hepatitis B virus, and coxsackievirus, most studies^12^, ^13^, ^16^ reported a weak or null association, and a few studies^11^, ^15^, ^18^ reported a significantly positive result. Therefore, whether maternal viral infection is an independent risk factor of CHD in offspring remains controversial. It should be noted that, so far, no any meta‐analysis has been conducted.

The present study represents, to our knowledge, the first meta‐analysis of risk of CHD associated with maternal viral infections. Our study has important strengths. This review is the most up to date on this subject. With the accumulating evidence and enlarged sample size, we have enhanced statistical power to provide more precise and reliable risk estimates. In our study, 52.9% of the studies included in this subgroup analysis had a large sample size (>500); all included studies were considered to be of higher methodologic quality. All of the original studies included focused on the link between maternal viral infection in early pregnancy and CHD, which, because the critical period of heart development is in early pregnancy,^29^ can help us to explain causality to some extent. Moreover, the association between maternal viral infections and CHD risk persists and remains statistically significant in sensitivity analysis on the basis of various exclusion criteria. The most relevant heterogeneity moderators have been identified by subgroup analysis.

Although our study suggested that the risk of CHD in offspring was significantly increased among mothers with viral infections, the reasons are not clear, which was rarely discussed in previous studies. Previous studies^30^ have shown that rubella virus, herpesvirus, and cytomegalovirus were human teratogens that could cause a spectrum of birth defects, including blindness, deafness, CHDs, mental retardation, and central nervous system complications, if the viral infection is acquired in the early months of pregnancy. However, our meta‐analysis did not find that mothers with herpesvirus and cytomegalovirus had a significantly increased risk of fetal CHD. In addition, the increased risk of CHD associated with maternal viral infections may be associated with drugs used after viral infections. An animal study showed that analgesics‐antipyretics had a teratogenic effect on fetal cardiovascular development.^31^ However, there is still no consensus with regard to the influence of analgesics‐antipyretics on humans. For example, some studies showed that the use of ibuprofen (an analgesic‐antipyretic) can decrease the incidence of CHD by decreasing the teratogenic effect of fever and influenza.^32^, ^33^ Additionally, some studies showed that exposure to erythromycin (an antibiotic) in early pregnancy was associated with a higher risk of CHD; however, other studies showed that there was no relationship.^34^, ^35^ The uncertainty of underlying mechanisms between maternal viral infections and CHD risk warrants further research.

Heterogeneity is often a concern in a meta‐analysis. In the present review, substantial heterogeneity was found among studies of maternal viral infections and CHD risk, which was not surprising given the differences in study population characteristics, study design types, ascertainment of viral infection, diagnostic method of CHD, adjustment for confounding factors, and study quality. We used subgroup analyses to explore the potential sources of heterogeneity on the basis of the above‐mentioned characteristics. As a result, whether the confounding factors were adjusted was identified as the most relevant heterogeneity moderator. So far, there has been a general consensus that CHD is a polygenic disease caused by both genetic and environmental factors. Many factors, including maternal socioeconomic situation, education, dietary pattern, diabetes mellitus, obesity, metabolic syndrome, alcohol consumption, smoking, and genetic background, have been reported to be associated with risk of CHD.^36^ Therefore, these factors should be controlled when evaluating the effect of maternal viral infections on fetal CHD. In our review, only 35.3% of included studies controlled for confounding factors when assessing the association between maternal viral infection and risk of CHD in offspring, which may lead to substantial heterogeneity when combining adjusted and unadjusted data for risk estimation. However, the results from our subgroup and sensitivity analyses were similar and robust, and the associations were neither significantly modified by study design types, ascertainment of virus infection, diagnosis method of CHD, whether the confounding factors were adjusted, or study quality nor substantially driven by any single study. A significantly positive association was observed in all subgroups, except in unadjusted data and studies with a quality score equal to 6 or 7.

### Limitations

Potential limitations of this study should be considered in future studies. First, in our review, we estimated the risk of total CHD associated with maternal viral infections, but we did not estimate the risk of specific CHD subtypes. In fact, during the design phase of our study, we did consider estimating the risk of specific CHD subtypes associated with maternal viral infections. However, all of the studies included did not provide data on specific subtypes of CHD. CHDs include many pathological types, such as atrial septal defects, ventricular septal defects, patent ductus arteriosus, tetralogy of Fallot, and so on. Future studies should focus on the association between maternal viral infection and specific CHD subtypes.

Second, although the present review did include studies published in Chinese or English, all included studies were from China, except one study from Canada, which may restrain the popularization and application of present findings. After repeated confirmation, our search strategy was correct. Therefore, additional research in other populations is warranted to generalize the findings.

Third, residual confounding is a concern. Uncontrolled or unmeasured risk factors have the potential to produce biases. Although restricting analysis to studies that have matched or adjusted confounding factors did not materially alter the combined risk estimate, the possibility cannot be ruled out that residual confounding affected the results because these factors do not explain all CHD risk.

Fourth, estimates were substantially heterogeneous across studies. Nevertheless, we were able to detect the major source of heterogeneity through the subgroup analysis and the sensitivity analysis. The sensitivity analysis that omitted one study at a time and calculated the combined OR for the remaining studies yielded consistent results. After subgroup analysis, the heterogeneity was obviously decreased. However, our estimates must be viewed with caution because of heterogeneity.

Fifth, the assessment of maternal viral infections and CHD diagnosis were different across studies, which may lead to classification bias. For example, in all included studies, 9 studies (52.9%) used laboratory testing to assess whether mothers were infected with a virus; while the remaining studies (47.1%) used questionnaires. Therefore, we cannot rule out the possibility that the mother's viral infection may be underreported. In addition, all included studies are case‐control studies, which may lead to recall bias. Furthermore, for risk estimates of CHD associated with specific viral infections, the results mainly relied on between 3 and 7 of the 17 total studies, so more studies should be included in future reviews to provide further support for our results. Last but not least, after reviewing the funnel plot, we can identify 4 of 17 papers outside the funnel, which means that around 25% of studies included had important publication bias. Therefore, potential publication bias could influence the findings.

## Conclusions

Our study, which includes a large number of participants, giving it sufficient statistical power, aimed to address the question of whether mothers who were infected with a virus in early pregnancy were at a higher risk of CHD in offspring compared with those without viral infections. Although the role of potential bias and evidence of heterogeneity should be carefully evaluated, our study indicated that maternal viral infections in early pregnancy are significantly associated with risk of CHD in offspring. However, the underlying mechanisms involved in the association between maternal viral infections and CHD risk are uncertain and require further study for elucidation. In the future, some large and multicenter prospective cohort studies will need to confirm our findings.

## Sources of Funding

Qin was supported by the Project Funded by Natural Science Foundation of Hunan Province (2018JJ2551), National Natural Science Foundation Program of China (81803313), Hunan Provincial Key Research and Development Program (2018SK2063), and New Teachers’ Scientific Research Driven Foundation of Central South University (502045001). The sponsor of the study had no role in study design, data collection, data analysis, data interpretation, or writing of the report.

## Disclosures

None.
